# Role of Hydrogen Sulfide and 3-Mercaptopyruvate Sulfurtransferase in the Regulation of the Endoplasmic Reticulum Stress Response in Hepatocytes

**DOI:** 10.3390/biom10121692

**Published:** 2020-12-18

**Authors:** Theodora Panagaki, Elisa B. Randi, Csaba Szabo

**Affiliations:** Chair of Pharmacology, Section of Medicine, University of Fribourg, CH-1700 Fribourg, Switzerland; elisa.randi@unifr.ch

**Keywords:** hydrogen sulfide, ER stress, mitochondria, autophagy, hepatic UPR

## Abstract

It is estimated that over 1.5 billion people suffer from various forms of chronic liver disease worldwide. The emerging prevalence of metabolic syndromes and alcohol misuse, along with the lack of disease-modifying agents for the therapy of many severe liver conditions predicts that chronic liver disease will continue to be a major problem in the future. Better understanding of the underlying pathogenetic mechanisms and identification of potential therapeutic targets remains a priority. Herein, we explored the potential role of the 3-mercaptopyruvate sulfurtransferase/hydrogen sulfide (H_2_S) system in the regulation of the endoplasmic reticulum (ER) stress and of its downstream processes in the immortalized hepatic cell line HepG2 in vitro. ER stress suppressed endogenous H_2_S levels and pharmacological supplementation of H_2_S with sodium hydrogen sulfide (NaHS) mitigated many aspects of ER stress, culminating in improved cellular bioenergetics and prevention of autophagic arrest, thereby switching cells’ fate towards survival. Genetic silencing of 3-MST or pharmacological inhibition of the key enzymes involved in hepatocyte H_2_S biosynthesis exacerbated many readouts related to ER-stress or its downstream functional responses. Our findings implicate the 3-MST/H_2_S system in the intracellular network that governs proteostasis and ER-stress adaptability in hepatocytes and reinforce the therapeutic potential of pharmacological H_2_S supplementation.

## 1. Introduction

Hydrogen sulfide (H_2_S), a gaseous endogenous biological mediator, plays an important role in the regulation of various mammalian cell functions. The main mammalian enzymes responsible for H_2_S biosynthesis are cystathionine-β-synthase (CBS), cystathionine-γ-lyase (CSE), and 3-mercaptopyruvate sulfurtransferase (3-MST) [1,2,3]. While the functional roles of CBS and CSE have been extensively investigated over the last decade and a half, the biological regulatory roles of the 3-MST/H_2_S system in health and disease are less understood [1,2,3,4,5,6]. 

Unresolved endoplasmic reticulum (ER) stress is now recognized as a pathogenic mechanism for hepatic injury and malfunction upon a variety of pathophysiological conditions, including metabolic syndrome. Shared among these seemingly different pathophysiological liver manifestations is the presence of intracellular or extracellular conditions that perturb calcium handling and the dynamics for protein folding and degradation. This response, in turn, generates a vicious cycle of persistent ER stress, which can exhaust the adaptive regenerative capacity of the organ to promote pro-inflammatory and pro-apoptotic signaling, ultimately favoring hepatic injury (which, depending on the underlying disease, can produce various functional and histopathological pictures ranging from hepatic dysfunction to hepatic scarring and hepatocellular carcinoma [7,8,9]. Pharmacological or therapeutic approaches that counteract ER stress may be useful to limit the progression of various forms of liver diseases [7,8,9].

The current study was designed to explore the potential role of the 3-MST/H_2_S system in the regulation of the cellular response under endoplasmic reticulum (ER) stress (hereafter occasionally referred to as stress conditions) in the immortalized hepatic cell line HepG2.

## 2. Materials and Methods 

### 2.1. Materials

Cell proliferation kit II (2,3-Bis-(2-methoxy-4-nitro-5-sulphophenyl)–2H–tetrazolium–5–carboxanilide) (XTT; Ref ID: 11465015001), Cell Proliferation 5-bromo-2′-deoxyuridine (BrdU) colorimetric ELISA kit (Ref ID: 11647229001), and Cytotoxicity Detection Kit^PLUS^ (lactate dehydrogenase (LDH); Ref ID: 4744934001) were purchased from Roche Diagnostics Ltd. (Sigma-Aldrich Chemie GmbH, Buchs, Switzerland). Agilent Seahorse XF Glycolytic Rate Assay (Ref ID: 103344-100) and Cell Mito Stress Test (Ref ID: 103015-100) kits, Seahorse XF-24 cell culture microplates, and the corresponding Seahorse XF assay media and calibrant solution were acquired from Agilent Technologies AG (Basel, Switzerland). The 3-MST inhibitor [10] 2-[(4-hydroxy-6-methylpyrimidin-2-yl)sulfanyl]-1-(naphthalen-1-yl)ethan-1-one (HMPSNE) was purchased from MolPort SIA, Riga, Latvia. Sodium hydrosulfide monohydrate (NaHS; ≥90%), Aminooxyacetic acid hemihydrochloride (AOAA; 98%), MISSION^®^ esiRNA human 3-MST (Ref ID: EHU086511) along with its corresponding universal negative control (Ref ID: SIC001), nuclease-free water, 7-azido-4-methylcoumarinv (AzMC), autophagy assay kit, and dimethyl sulfoxide (DMSO; anhydrous, ≥99.9%) were obtained from Sigma-Aldrich Chemie GmbH. Bovine serum albumin (BSA) was acquired from Cell Signaling Technology (CST; Bioconcept AG, Allschwil, Switzerland). Other materials and reagents for cell culture, cell transfection, protein concentration estimation, Western blotting, and live-cell labeling were purchased from Thermo Fisher Scientific (Basel, Switzerland), unless otherwise stated. 

### 2.2. Cell Culture

The human hepatoma HepG2 (ATCC^®^ HB-8065™) cell line was acquired from LGC Standards (Molsheim, France). The non-tumorigenic HepG2 cells were cultured in DMEM basal medium supplemented with 1X Glutamax™, 10% heat-inactivated fetal bovine serum (FBS), 100 IU/mL of penicillin, and 100 µg/mL of streptomycin. The cells were maintained at 37 °C in a humidified incubator with 5% CO_2_ and 95% air. Culture medium was renewed twice per week. Cells were sub-cultured when 80–90% confluent and grown up to passage 10 to avoid loss of hepatic-specific functions. Propagating cells were seeded at a ratio of 1:10 or at the desired cell density for each assay. Viable cells were quantified with the automated R1 Cell Counter (Olympus Schweiz AG, Volketswil, Switzerland). 

### 2.3. Cell Treatments

Thapsigargin (TG), tunicamycin (TM), and HMPSNE were reconstituted in 100% DMSO at a stock concentration of 1 mM, 10 mg/mL, and 0.5 M, respectively. Stock solutions were aliquoted and stored at −20 °C until further use. All aliquots were thawed once and serially diluted in the serum-free basal growth medium to final desired working concentrations for each experimental setup. NaHS and AOAA were maintained in powdered, desiccated form at 4 °C and reconstituted in serum-free basal medium to an initial concentration of 10 mM and 100 mM immediately before use. Stock solutions were serially diluted to the desired final working concentrations of 50–600 µM or of 30–100 µM using the corresponding medium. All cell treatments were conducted at least in duplicate per assay per experimental setup.

### 2.4. Cell Transfections

HepG2 were seeded at a density of 1 × 10^6^ cells at 95% viability in Corning^®^ cell culture T-75 flasks that corresponded to 70–80% confluency the following day. Short interfering RNA (siRNA) were delivered to the cells using the Lipofectamine™ 2000 reagent, as per the manufacturer’s protocol. Opti-MEM^®^ I Reduced Serum Medium replaced basal growth medium during the transfection and used for diluting Lipofectamine™ 2000 and RNA oligomers prior to complex formation. Twenty-four hours following the initiation of the transfection, cells were harvested and seeded at the corresponding micro-plates and culture vessels for each assay. Cell treatments were conducted 56 h following the initiation of the transfection. Gene silencing efficiency was monitored with Western blotting.

### 2.5. Assessment of Cell Viability and Proliferation

Cell Proliferation kit II (XTT), Cell Proliferation ELISA BrdU (colorimetric) kit and Cytotoxicity Detection Kit^PLUS^ (LDH) were respectively used for the quantification of hepatic cell viability, proliferation and necrosis. The assays were formatted in Corning^®^ Costar^®^ TC-Treated, flat-bottom, transparent 96-well microplates. HepG2 cells were seeded at a density of 2 × 10^4^ cells at 95% viability per well in total of 100 µL for 24 h. They were serum-starved for 8 h. Afterward, they were stressed with 10, 100, and 1000 nM thapsigargin for 16 h and assayed with the Cell proliferation kit II and Cytotoxicity Detection Kit^PLUS^, as per our previously published methodology [11]. Upon the selection of the optimal ER stressor concentration, cells were treated with vehicle control or 100 nM thapsigargin in the presence or absence of various concentrations NaHS (0–600 µM), HMPSNE (0–100 µM) or AOAA (0–100 µM) for 16 h. Cells were then assayed with the Cell Proliferation Kit II and Cell Proliferation ELISA BrdU kit. Vehicle control treatment refers to the maximum concentration of DMSO used that was 0.1%. 

### 2.6. Assessment of Cellular Bioenergetics

Extracellular flux (XF) analysis deployed for real-time quantification of oxygen consumption rate (OCR) and the proton efflux rate (PER) with the Agilent Seahorse XF Cell Mito Stress Test kit and Agilent Seahorse XF Glycolytic Rate Assay kit, respectively. Both assays were formatted in Seahorse XF24 cell-culture microplates and performed as previously described [11]. HepG2 cells were seeded at a density of 2 × 10^4^ cells per well in a total of 200 µL for 24 h. Cells were serum-starved for 8 h and treated with vehicle control, 100 nM thapsigargin, or 1 µg/mL tunicamycin for 16 h prior to the initiation of measurements. To assess the effect of the pharmacological regulation of the intracellular H_2_S levels, cells were co-treated with 100 µM NaHS, 100 µM HMPSNE, or 30 µM AOAA.

### 2.7. Live-Cell Labelling for Autophagy and Endogenous H_2_S Quantification

The autophagy assay kit and AzMC fluorogenic probe were accordingly used for real-time quantification of autophagosome formation and endogenous H_2_S levels. Both assays were formatted in Corning^®^ Costar^®^ TC-Treated, optical-bottom, black, 96-well microplates. HepG2 cells were seeded at a density of 1 × 10^4^ cells per well in a total of 100 µL for 24 h. Cells were serum-starved for 8 h and treated with vehicle control or 100 nM thapsigargin and 100 µM NaHS for 16 h. After the treatments, spent medium was discarded and the cells were incubated with 1X of the Autophagosome Detection Reagent in the kit-provided stain buffer for 30 min at 37 °C in a humidified incubator with 5% CO_2_ and 95% air. Cells were washed thrice and the fluorescence signal of the Autophagosome Detection Reagent was read with an Infinite^®^200 PRO microplate reader at λ_excitation_ = 360 nm and λ_emission_ = 520 nm. Alternatively, cells were incubated with 100 µM of the AzMC probe in pre-warmed Gibco™ Hanks’ Balanced Salt Solution (HBSS) with calcium and magnesium for 1 h. The AzMC fluorescence signal was read with an Infinite^®^200 PRO microplate reader at λ_excitation_ = 365 nm and λ_emission_ = 450 nm and normalized to the corresponding protein content of each microculture. Representative images of each cell labeling were captured under the Olympus CKX53 inverted fluorescent microscope with a DAPI channel. 

### 2.8. Live-Cell Labeling for Mitochondrial Superoxide

The MitoSOX™ Red mitochondrial superoxide indicator [12] was used for real-time quantification of mitochondrial oxidative stress following the cell handling and treatments described in the previous subsection. The fluorogenic probe was reconstituted 100% DMSO at a stock concentration of 5 mM, aliquoted and stored as per manufacturer’s guidelines. Stock solutions were diluted in pre-warmed HBSS with calcium and magnesium to the final working concentration of 5 µM. Cells were loaded with the working solution and incubated for 10 min at 37 °C in a humidified incubator with 5% CO_2_ and 95% air. Cells were washed thrice and the fluorescent signal of MitoSOX™ probe was read with an Infinite^®^200 PRO microplate reader at λ_excitation_ = 510 nm and λ_emission_ = 580 nm. Acquired data were normalized to the corresponding total cell number with Janus green staining (Abcam, Cambridge, UK). Janus green staining was performed according to the manufacturer’s protocol. 

### 2.9. Protein Extraction and Western Blotting

HepG2 cells were seeded at a density of 5 × 10^5^ cells in Corning^®^ cell culture T-25 flasks. Cells were serum-starved for 8 h and treated with vehicle control, 100 nM thapsigargin, or 1 µg/mL tunicamycin for 16 h. To assess the effect of the pharmacological regulation of the intracellular H_2_S levels, cells were co-treated with 100 µM NaHS, 100 µM HMPSNE, or 30 µM AOAA. Following the incubation with the treatments, the cells were washed twice with ice-cold 1X phosphate-buffered saline (PBS) and harvested in 1X PathScan^®^ Sandwich ELISA cell lysis buffer supplemented with protease/phosphatase inhibitor cocktail (1X). After two freeze/thaw cycles, the whole-cell lysate was collected and sonicated for 1 min (5 s ON/5 s OFF-6 cycles). The total protein was extracted by centrifugation at 16,000× *g* at 4 °C for 15 min. The Pierce™ Coomassie Plus (Bradford) protein assay was deployed for the estimation of the protein concentration of the samples. Protein samples of whole-cell lysate were aliquoted and stored at −80 °C until processed for Western blotting, in accord with our previously published protocol [13]. The primary antibodies used in the Western blotting experiments are summarized in Table 1. 

### 2.10. Statistics

All the results were expressed as mean ± standard error (SEM) of at least three independent experiments. Assumption of normality was examined with D’Agostino–Pearson’s K-squared and Shapiro–Wilk tests. Differences among means were considered significant if *p* ≤ 0.05. Data were analyzed with one- or two-way ANOVA analysis, followed by post-hoc Bonferroni’s multiple-comparison *t*-tests to identify differences among experimental groups and effects of the co-treatments of H_2_S donors and inhibitors under control and ER-stressed conditions. Statistical calculations were performed in GraphPad Prism 8 (GraphPad Software Inc., San Diego, CA, USA) for Mac OS X software.

## 3. Results

### 3.1. H_2_S Donation Rescues Thapsigargin-Induced Cell-Growth Arrest

**Figure 1:**
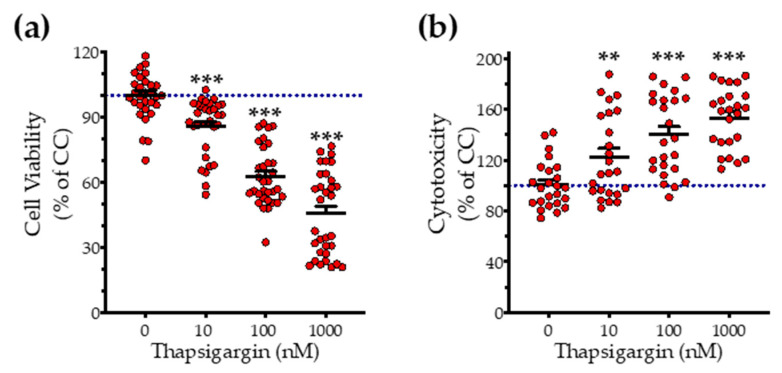
**Concentration-dependent effect of thapsigargin on hepatic-cell viability.** HepG2 cells were serum-starved for 8 h and treated with 0, 10, 100 and 1000 nM of thapsigargin for 16 h. Micro-cultures were assayed for the XTT conversion (**a**) to assess cell viability. Cell supernatant was collected and processed for the LDH activity (**b**) to determine cytotoxicity. Each line represents mean ± SEM from four independent experiments. Data are expressed as a percentage of the control conditions (CC). CC refer to vehicle-treated cells that correspond to 0 nM of the ER stressor and 0.1% DMSO (the vehicle). ** *p* ≤ 0.01; *** *p* ≤ 0.001, when compared to CC.

**Figure 2:**
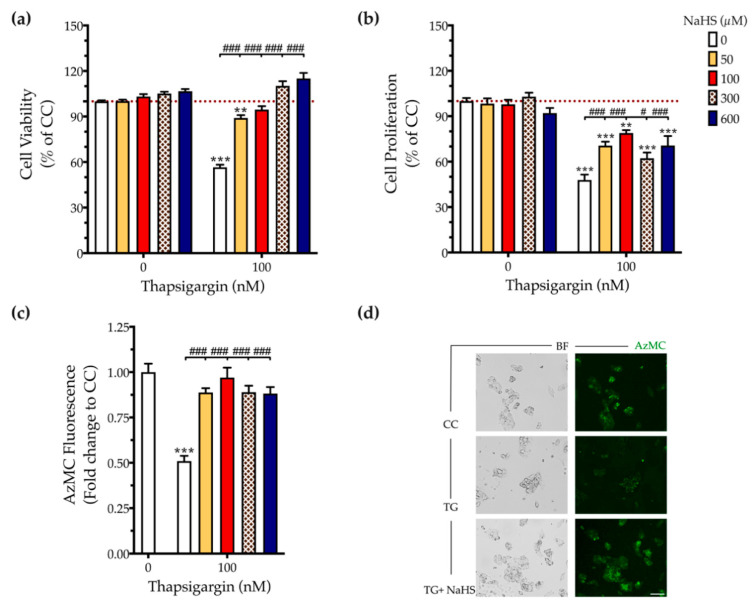
**NaHS co-treatment normalizes the endogenous H_2_S levels and rescues hepatic-cell viability and proliferation during chronic ER stress.** HepG2 cells were serum-starved for 8 h and treated with the vehicle or 100 nM of thapsigargin (TG), in the presence or absence of NaHS for 16 h. Micro-cultures were assayed for the XTT conversion (**a**) and BrdU incorporation (**b**) to assess cell viability and proliferation, respectively. Alternatively, micro-cultures were labeled with 100 µM AzMC to quantify the endogenous H_2_S levels (**c**). Representative pictures of the AzMC signal were captured with an Olympus CKX53 inverted microscope at 10× magnification; the left sub-panel corresponds to the bright-field channel (BF) for whole-cell imaging, whereas the right sub-panel to the DAPI fluorescent channel for AzMC imaging (**d**). Each bar represents mean ± SEM from four independent experiments. Data are expressed as a percentage or fold change of the control (vehicle-treated) conditions (CC). ** *p* ≤ 0.01; *** *p* ≤ 0.001, when compared to CC; ^#^ *p* ≤ 0.05; ^###^ *p* ≤ 0.001, when compared to the TG-treated HepG2 cells. Scale bar: 20 µm.

First, we addressed the effect of thapsigargin in hepatic cell physiology for determining the optimal ER stressor concentration. As shown in Figure 1 and Figure 2, XTT conversion and BrdU incorporation decreases while the LDH release rises in the supernatant in response to thapsigargin, indicating cell-growth arrest and cell death under conditions of ER stress. The intermediate concentration of the ER stressor, i.e., 100 nM produced a 40% reduction in cell viability (Figure 1a and Figure 2a) and proliferation (Figure 2b) that corresponds to half-fold increase in cytotoxicity (Figure 1b). Therefore, we have selected the 100 nM concentration of thapsigargin for the subsequent experiments. Thapsigargin at 100 nM significantly suppressed cellular H_2_S levels, evidenced by an approximately 50% suppression in the fluorescent signal of the H_2_S probe, AzMC (Figure 2c,d). H_2_S donation with NaHS increased the levels of this gaseous signaling molecule, with the concentration of 100 µM reaching the control (normal) levels, as shown in Figure 2c. NaHS also produced an amelioration of the aberrant hepatic cell viability and proliferation, as illustrated in Figure 2a,b. Among the concentrations evaluated, 100 µM of NaHS was the most effective to restore cell viability and proliferation under ER stress conditions, without affecting basal cell growth (in the absence of the ER stressor) (Figure 2). Thus, we have selected the concentration of 100 µM of the H_2_S donor for our subsequent experiments.

### 3.2. H_2_S Donation Ameliorates the Hepatic Unfolded Protein Response and Inhibits Autophagy Arrest

**Figure 3:**
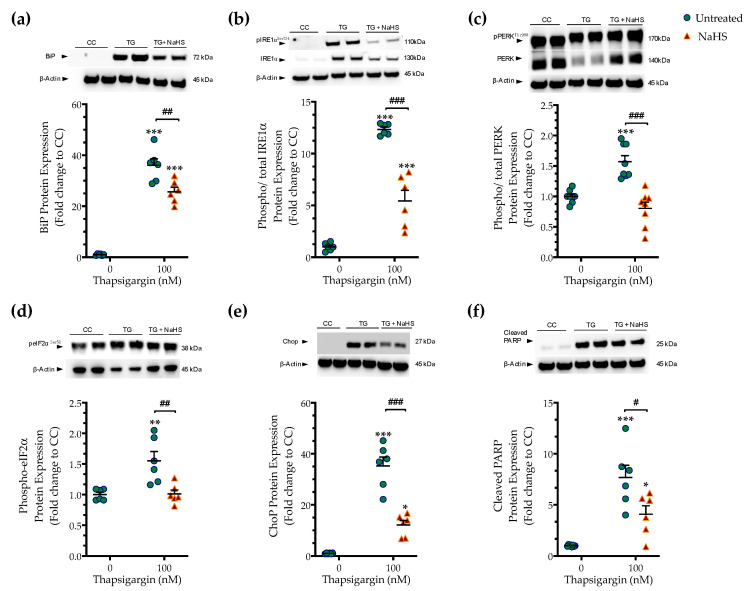
**NaHS co-treatment ameliorates the hepatic unfolded protein response (UPR).** HepG2 cells were serum-starved for 8 h and treated with the vehicle or 100 nM of thapsigargin (TG), in the presence or absence of 100 µM NaHS for 16 h. Cells were harvested and the extracted protein was processed for Western blotting analysis of the expression levels of BiP (**a**), activating phosphorylation of IRE1α at the serine residue 724 (Ser724) (**b**), the activating phosphorylation of PERK at the threonine residue 980 (Thr980) (**c**), the inhibitory phosphorylation of eIF2α at Ser51 (**d**), Chop (**e**), and of cleaved PARP (**f**). Each graph line represents mean ± SEM from three independent experiments. Data are expressed as a fold change of the control (vehicle-treated) conditions (CC). * *p* ≤ 0.05, ** *p* ≤ 0.01; *** *p* ≤ 0.001 compared to CC; ^#^ *p* ≤ 0.05, ^##^ *p* ≤ 0.01, ^###^ *p* ≤ 0.001 compared to TG-treated HepG2 cells. For the abbreviations used, please refer to the main text.

Next, we tested whether the cytoprotective effects of NaHS co-treatment could affect the regulation of the unfolded protein response (UPR) under conditions of persistent ER stress. We harvested cells that had been previously treated with the vehicle or 100 nM thapsigargin in the presence or absence of 100 µM NaHS. The extracted protein samples were then processed for Western blotting of key effectors and mediators of the UPR. Thapsigargin elicited a more than 30- and 10-times increase in the expression levels of the binding immunoglobulin protein (BiP) (Figure 3a) and of the activating phosphorylation of inositol-requiring enzyme-1α (IRE1α) (Figure 3b), respectively. It also doubled the phosphorylated levels of protein kinase R (PKR)-like endoplasmic reticulum kinase (PERK) (Figure 3c) and its downstream target for global cell translation-the eukaryotic initiation factor 2 (eIF2) *α* subunit (Figure 3d), indicating the induction of ER stress response [14]. 

Thapsigargin treatment induced a 30-fold rise in the expression levels of the C/EBP-homologous protein (Chop (Figure 3e), a protein that is recognized for its signaling role in the proapoptotic phase of the UPR (“terminal UPR”) [14]. The latter mechanism is further supported by the aberrant cleavage of poly [ADP-ribose] polymerase (PARP) 1, as shown in Figure 3f. NaHS co-treatment reduces the ectopic BiP expression and IRE1*α* activation (Figure 3a,b), although their levels have remained significantly elevated when compared to control conditions (*p* ≤ 0.001). NaHS co-treatment also restored PERK signaling, evidenced by the normalization of the activating phosphorylation of PERK (Figure 3c), of the inhibitory phosphorylation of eIF2*α* (Figure 3d) and of the intracellular Chop content (Figure 3e). The latter effect also correlated with a significant prevention of thapsigargin-induced, ER-stress-associated PARP1 cleavage (Figure 3f). These findings indicate that H_2_S donation ameliorated the activation and expression patterns of effectors proteins of the ER stress response that downstream shifted the UPR towards a pro-survival outcome.

**Figure 4:**
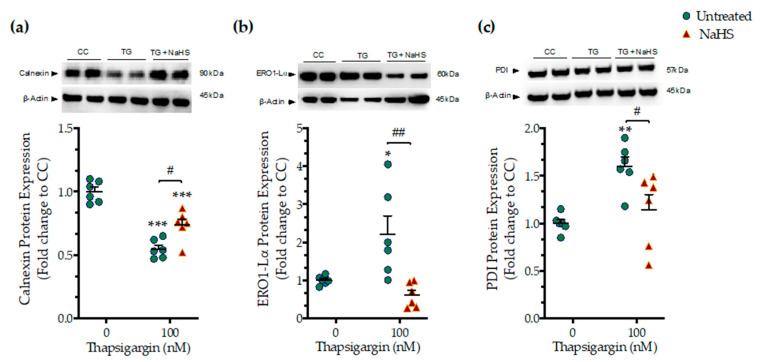
**NaHS co-treatment partially counteracts the dysregulation of the expression of local chaperones for the ER folding machinery.** HepG2 cells were serum-starved for 8 h and treated with the vehicle or 100 nM of thapsigargin (TG), in the presence or absence of 100 µM NaHS for 16 h. Cells were harvested and the extracted proteins were processed for Western blotting analysis of the expression levels of calnexin (**a**), ER oxidoreductase 1α (ERO1-Lα) (**b**), and protein disulfide isomerase (PDI) (**c**). Each graph line represents mean ± SEM from three independent experiments. Data are expressed as a fold change of the control (vehicle-treated) conditions (CC). * *p* ≤ 0.05, ** *p* ≤ 0.01; *** *p* ≤ 0.001 compared to CC; ^#^ *p* ≤ 0.05, ^##^ *p* ≤ 0.01 compared to TG-treated cells.

We next assessed whether the NaHS co-treatment could affect the ER folding machinery during ER stress. In order to examine this question, we quantified the expression levels of the protein quality-control chaperones–calnexin (Figure 4a), ER oxidoreductase 1*α* (ERO1-L*α*) (Figure 4b), and protein disulfide isomerase (PDI) (Figure 4c). Chronic thapsigargin treatment significantly down-regulated calnexin while it up-regulated ERO1-L*α* and PDI expression. NaHS co-treatment rescued the suppressed calnexin expression and normalized ERO1-L*α* and PDI levels, as shown in Figure 4. These biochemical observations suggest that the exogenous H_2_S donation can preserve essential proteostatic mechanisms [14] under the conditions of persistent ER stress.

**Figure 5:**
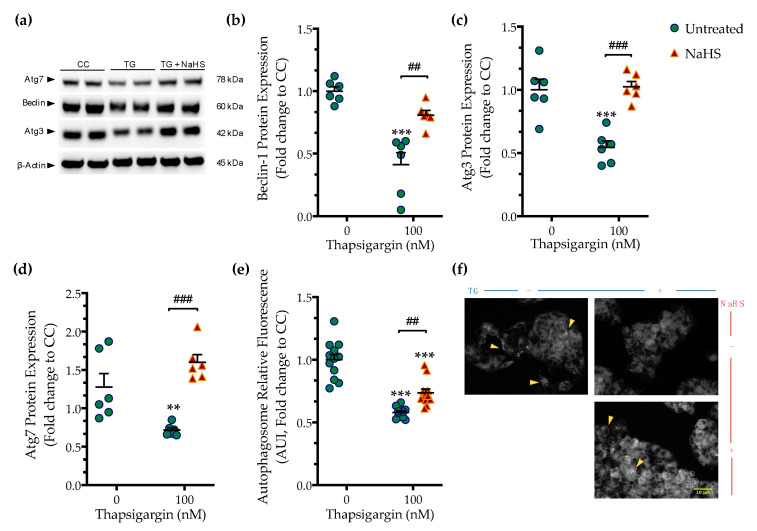
**NaHS co-treatment rescues the arrested autophagy following chronic ER stress.** HepG2 cells were serum-starved for 8 h and treated with the vehicle or 100 nM of thapsigargin (TG), in the presence or absence of 100 µM NaHS for 16 h. Cells were harvested, and the extracted protein was processed for Western blotting analysis of the expression levels of beclin-1 (**a**,**b**), Atg3 (**a**,**c**), and Atg7 (**a**,**d**). Alternatively, cells were incubated with the Autophagosome Detection Reagent working solution for 30 min at 37 °C with a humidified incubator with 5% CO_2_ and 95% air. Cells were washed thrice and the fluorescence signal was read with an Infinite^®^200 PRO microplate reader (**e**). Representative images of the labeling were captured under an Olympus CKX53 inverted fluorescent microscope with a DAPI channel (**f**). Autophagy is indicated by bright dot staining of the autophagic vacuoles (yellow arrowheads). Data are expressed as a fold change of the control (vehicle-treated) conditions (CC). ** *p* ≤ 0.01, *** *p* ≤ 0.001 compared to CC; ^##^ *p* ≤ 0.01, ^###^ *p* ≤ 0.001 compared to TG-treated cells. For the abbreviations used, please refer to the main text. Scale bar: 10 µm.

Perturbation of ER calcium by thapsigargin hampers autophagosome biogenesis and its fusion with the lysosomes precludes macroautophagy (hereinafter referred to as autophagy) that can, in turn, elicit and/or exacerbate ER stress [15]. Accordingly, we observed that chronic thapsigargin treatment diminished the expression of the autophagy-related (Atg) proteins, beclin-1, Atg3, and Atg7 (Figure 5) that are known to orchestrate the formation, elongation and maturation of the autophagosome [16]. 

Deficiency in Atg components coincided with a significant reduction in the fluorescence signal of the autophagosome probe that corresponded to a decreased number of double-membrane vesicles in the cells, as shown in Figure 5. This observation pointed towards an autophagic arrest under conditions of chronic ER stress. Co-treatment with NaHS normalized the expression of beclin-1 (Figure 5a,b), Atg3 (Figure 5a,c), and Atg7 (Figure 5a,d). It additionally attenuated the decreased intercellular content of fluorescent-tagged vesicles, as shown in Figure 5e,f. These findings demonstrate that the pharmacological restoration of the suppressed intracellular H_2_S with the fast-releasing H_2_S donor–NaHS can restore the availability of autophagy components and rescue the arrested formation of autophagosomes during ER stress.

### 3.3. H_2_S Donation Restores Cellular Bioenergetics and Counteracts Mitochondrial Stress during ER Stress

**Figure 6:**
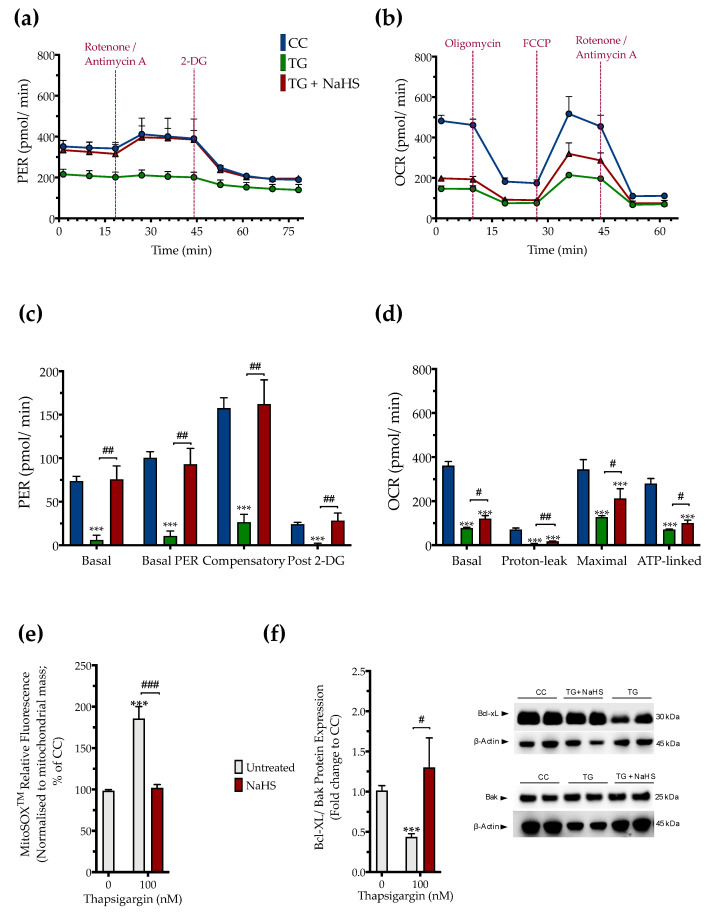
**NaHS improves cellular bioenergetics and reduces mitochondrial oxidant production during ER stress.** HepG2 cells were serum-starved for 8 h and treated with the vehicle or 100 nM of thapsigargin (TG), in the presence or absence of 100 µM NaHS for 16 h. We then monitored (**a**,**c**) the oxygen consumption rate (OCR) over the sequential injection of oligomycin (1 µM; ATP synthase inhibitor), FCCP (1.5 µM; mitochondrial uncoupler) and of rotenone + antimycin A (0.5 µM; Complex I and III inhibitors) and (**b**,**d**) the proton efflux rate (PER) over the sequential injection of rotenone + antimycin A and 2-deoxy-D-glucose (20 mM) with the Seahorse XFe24 Extracellular Flux Analyzer. We have also quantified (**e**) the relative fluorescence signal of MitoSOX™ red mitochondrial superoxide indicator and (**f**) the relative expression of Bcl-xL and Bak proteins. Each graph line and bar represent mean ± SEM from four independent experiments. Data are expressed as a fold change of the control (vehicle-treated) conditions (CC). *** *p* ≤ 0.001 compared to CC; ^#^ *p* ≤ 0.05, ^##^ *p* ≤ 0.01, ^###^ *p* ≤ 0.001 compared to the TG-treated cells.

The impaired cellular bioenergetics (as evidenced by a decreased basal OCR and PER) of the thapsigargin-treated HepG2 cells (compared to the control, unstressed conditions) suggests that persistent ER stress hindered hepatic oxidative phosphorylation and glycolysis to suppress both aerobic and anaerobic bioenergetic function, respectively. These alterations in cellular bioenergetic function correlated with a significant increase in the relative fluorophore intensity of the MitoSOX™ Red reagent, indicating increased mitochondrial oxidative stress (mitochondrial superoxide generation) by the dysfunctional mitochondria as shown in Figure 6. 

Pharmacological restoration of the suppressed endogenous H_2_S levels with NaHS partially protected against the ER-stress-associated decline in the various parameters for mitochondrial bioenergetics (Specifically, NaHS co-treatment improved the glycolytic parameters (Figure 6a,c) to a higher extent than its effect on the parameters of mitochondrial oxidative phosphorylation. That observation demonstrated a more pronounced beneficial effect of the exogenous H_2_S donation on the regulation of anaerobic respiration (as compared to aerobic) during ER stress. NaHS co-treatment also normalized the up-regulated superoxide signal (Figure 6e) and the decreased ratio of the pro-survival Bcl-xL to the pro-apoptotic Bak (Figure 6f) under conditions of persistent ER stress. The latter signaled for the ability of NaHS co-treatment to improve mitochondrial function and abrogate the activation of the intrinsic apoptotic pathway, respectively.

### 3.4. Effect of ER Stress on the Expression of Various H_2_S-Synthesizing and -Degrading Enzymes

As introduced earlier, CBS, CSE and 3-MST are the three principal enzymes generating H_2_S in mammalian cells. All three enzymes are abundantly expressed in the liver tissue and in hepatocytes [1,2,3], raising the possibility that these enzymes may become affected by ER stress and/or that the H_2_S produced endogenously by these enzymes may affect the progression of the ER stress response. 

**Figure 7:**
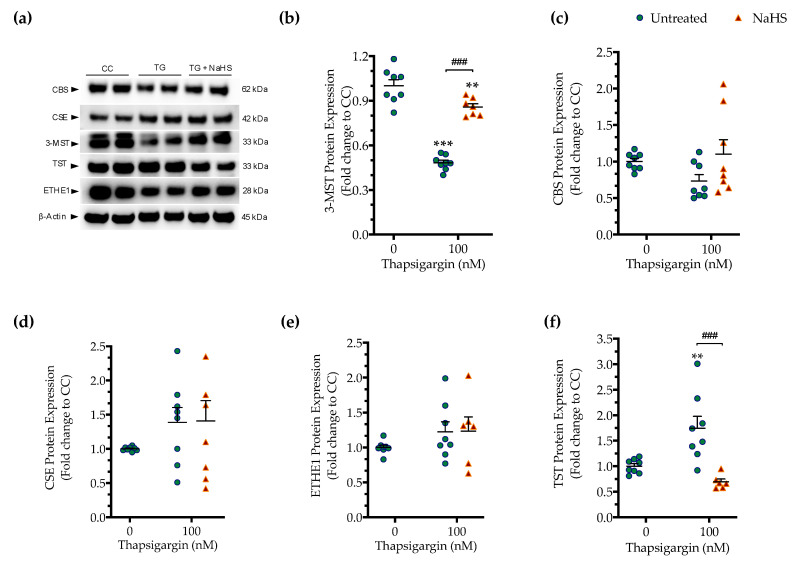
**Hepatic persistent ER stress selectively down-regulates the expression of 3-MST enzyme for H_2_S synthesis; such an effect is reversible upon the exogenous donation of H_2_S.** HepG2 cells were serum-starved for 8 h and treated with the vehicle or 100 nM of thapsigargin (TG), in the presence or absence of 100 µM NaHS for 16 h. Subsequently, cells were harvested and the extracted protein was processed for Western blotting analysis of 3-MST (**a**,**b**), CBS (**a**,**c**), CSE (**a**,**d**), ETHE1 (**a**,**e**), and TST (rhodanese) (**a**,**f**) enzymes. Each graph line represents mean ± SEM from four independent experiments. Data are expressed as a fold change of the control (vehicle-treated) conditions (CC). ** *p* ≤ 0.01 and *** *p* ≤ 0.001 compared to CC; ^###^ *p* ≤ 0.001 compared to the TG-treated cells. For abbreviations used, please refer to the main text.

First, we determined if the expression of the three principal H_2_S producing enzymes is affected by chronic ER stress. Persistent ER stress with 100 nM thapsigargin induced a significant downregulation of 3-MST, while the protein levels of CBS and CSE remained unaltered (Figure 7). The expression of the H_2_S-metabolizing enzyme ETHE1 did not significantly change under conditions of chronic ER stress (Figure 7e). However, the expression of thiosulfate sulfurtransferase (TST; also known as rhodanese) significantly increased in response to thapsigargin treatment (Figure 7f). Interestingly and unexpectedly, NaHS co-treatment restored 3-MST and TST expression during ER stress, predicting a possible feedforward or feedback mechanism between intracellular H_2_S levels, 3-MST and rhodanese (Figure 7). 

### 3.5. Effect of 3-MST Silencing on the Development of the ER Stress Response and on the Effect of H_2_S Donation

**Figure 8:**
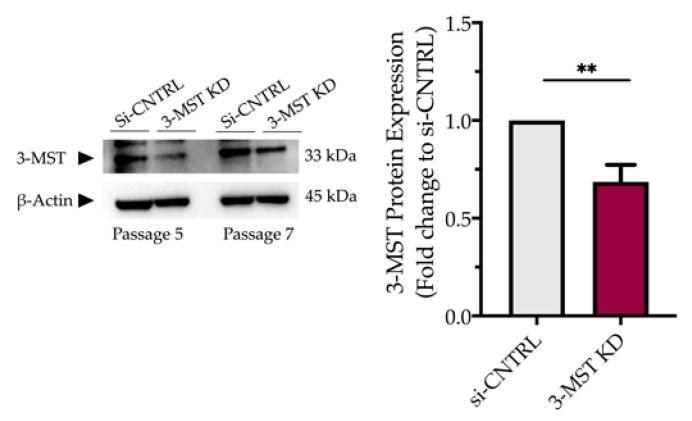
**Partial efficacy of 3-MST silencing using siRNA.** Quantification of 3-MST protein levels and representative blot pictures following RNA interference. Each bar represents mean ± SEM from four independent cell transfections. Data are expressed as a fold change of the transfection negative control (si-CNTRL); ** *p* ≤ 0.01.

**Figure 9:**
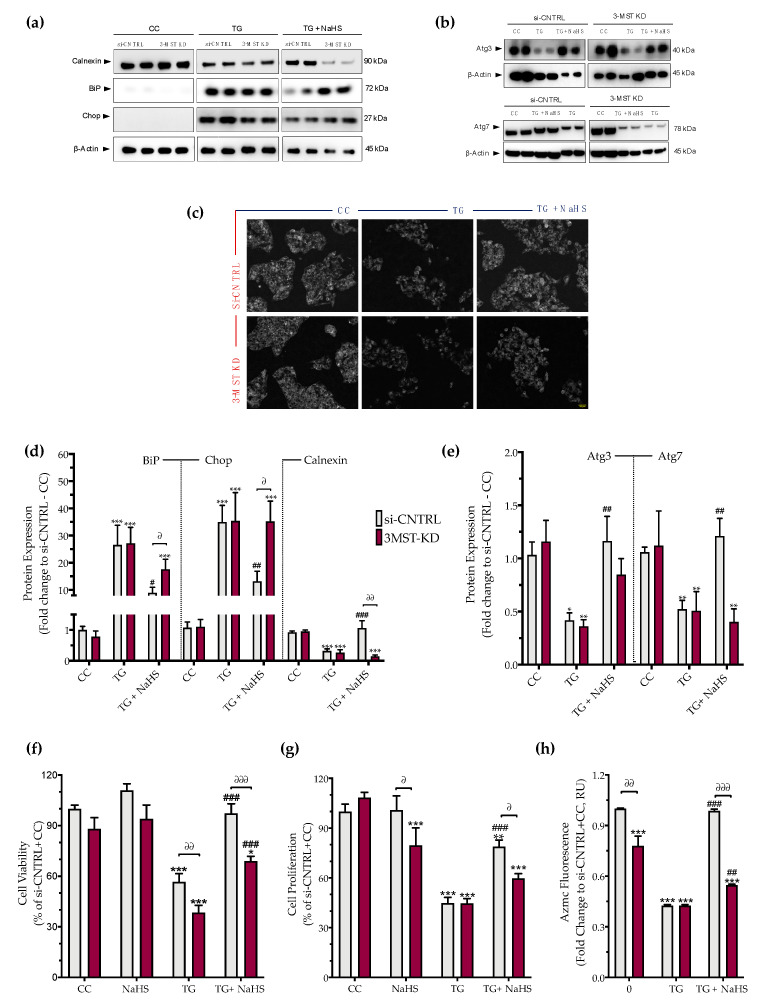
**3-MST silencing attenuates the restorative effects of the H_2_S donor NaHS on the ER stress response and autophagy.** HepG2 cells previously subjected to 3-MST silencing (knockdown (KD)) or sham-silencing (si-CNTRL) were serum-starved for 8 h and treated with the vehicle or 100 nM of thapsigargin (TG), in the presence or absence of 100 µM NaHS for 16 h. Whole-cell lysate was collected and processed for protein expression of the UPR sensor–BiP, the UPR apoptotic mediator–Chop, the ER chaperone–calnexin (**a**,**d**), and of the autophagy-related proteins Atg3 and Atg7 (**b**,**e**). Representative images of the cells previously labeled with the Autophagosome Detection probe were captured under the Olympus CKX53 inverted fluorescent microscope with a DAPI channel. Autophagy is indicated by bright dot staining of autophagic vacuoles (**c**). Micro-cultures were assayed for XTT conversion (**f**) BrdU incorporation (**g**), and AzMC fluorochrome intensity (**h**) to assess cell viability, cell proliferation, and intracellular H_2_S levels, respectively. Each bar represents mean ± SEM from three and four independent experiments for Western blotting and cell-physiology studies, respectively. Data are expressed as a percentage of the transfection negative control, control (vehicle-treated) conditions (si-CNTRL+CC). * *p* ≤ 0.05, ** *p* ≤ 0.01 and *** *p* ≤ 0.001 compared to corresponding CC; ^#^ *p* ≤ 0.05, ^##^ *p* ≤ 0.01 and ^###^ *p* ≤ 0.001 compared to the corresponding TG-treated cells; ^∂^ *p* ≤ 0.05, ^∂∂^ *p* ≤ 0.01 and ^∂∂∂^ *p* ≤ 0.001 compared to the corresponding 3-MST KD treatment group.

**Figure 10:**
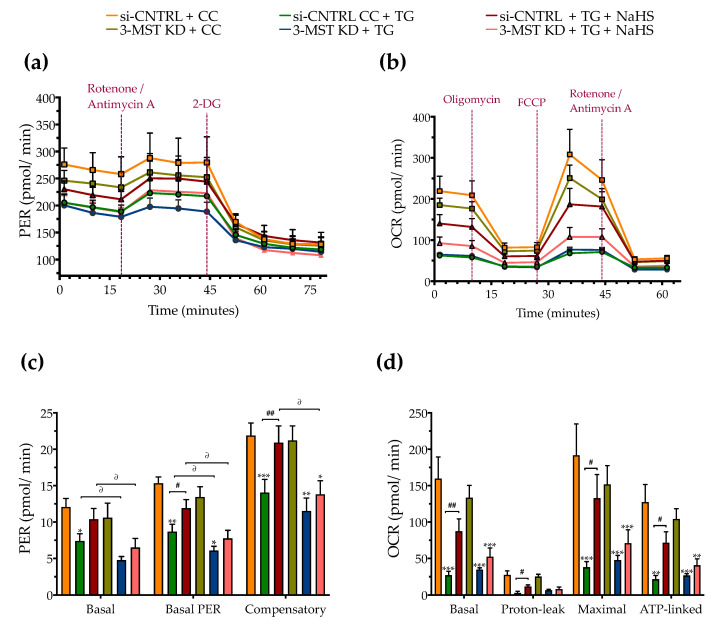
**Effect of 3-MST silencing (knockdown; KD) on cellular bioenergetic function during ER stress.** HepG2 cells previously subjected to 3-MST silencing (knockdown (KD)) or sham-silencing (si-CNTRL) were serum-starved for 8 h and treated with the vehicle or 100 nM of thapsigargin (TG), in the presence or absence of 100 µM NaHS for 16 h. We then monitored the proton efflux rate (PER) over the sequential injection of rotenone + antimycin A and 2-deoxy-D-glucose (20 mM) (**a**,**c**) and the oxygen consumption rate (OCR) over the sequential injection of oligomycin (1 µM; ATP synthase inhibitor), FCCP (1.5 µM; mitochondrial uncoupler) and of rotenone + antimycin A (0.5 µM; Complex I and III inhibitors) (**b**,**d**) with the Seahorse XFe24 Extracellular Flux Analyzer for assessing glycolysis and oxidative phosphorylation, respectively. Each graph line and bar represent mean ± SEM from four independent experiments. Data are expressed as a fold change of the sham-transfected control, vehicle-treated control conditions (si-CNTRL + CC). * *p* ≤ 0.05, ** *p* ≤ 0.01, and *** *p* ≤ 0.001 compared to corresponding CC; ^#^ *p* ≤ 0.05 and ^##^ *p* ≤ 0.01, compared to the corresponding TG-treated cells; ^∂^ *p* ≤ 0.05 compared to the corresponding 3-MST KD treatment group.

Partial silencing of the 3-MST enzyme (Figure 8) did not significantly affect baseline cellular parameters. This was evidenced by the unaltered levels of the UPR signaling nodes-BiP, Chop, and calnexin (Figure 9a,d), and of the expression levels of autophagy-related markers-Atg3 and Atg7 (Figure 9b,e). Similarly, mitochondrial respiratory activity (Figure 10), cell proliferation (Figure 9g), and cell viability (Figure 9f) remained unaltered between the sham-transfected and 3-MST silenced cells under baseline (non-ER-stressed) conditions. 

3-MST silencing did not deteriorate the UPR outcome (Figure 9a,d), the arrested autophagosome formation (Figure 9b–e), or the aberrant parameters of oxidative phosphorylation (aerobic respiration) (Figure 10b,d). It did not affect the thapsigargin-induced suppression of cell proliferation (Figure 9g). 

However, 3-MST silencing exacerbated the degree by which chronic ER stress suppressed cellular bioenergetic function. In particular, in the 3-MST-silenced cells, chronic thapsigargin treatment potentiated a more pronounced suppression of basal glycolytic rate and basal PER (Figure 10c). The impaired mitochondrial function was also evidenced by a more significant deterioration of the decreased XTT conversion for cell viability in the 3-MST silenced cells than in the sham-silenced controls after persistent ER stress (Figure 9f). 

Interestingly, the beneficial effects of NaHS co-treatment on the regulation of the UPR, autophagy and cell bioenergetics during persistent ER stress were no longer apparent in the cells with 3-MST silencing (in contrast to the efficacy of the H_2_S donor in the sham-silenced control cells), as shown in Figure 9 and Figure 10. These findings may account for a threshold level of H_2_S that exogenous H_2_S donation needs to be reached during ER stress in order to exert beneficial effects, and when one of the principal sources of endogenous H_2_S generation is impaired, this threshold may not be achieved with 100 µM NaHS. Consistent with this hypothesis, quantification of AzMC fluorescence signal revealed that the 3-MST knockdown significantly decreased the H_2_S levels at basal (non-ER stressed) conditions and partially counteracted the restoration of H_2_S levels by the exogenous H_2_S donation under conditions of persistent ER stress, as shown in Figure 9h.

### 3.6. Effect of Pharmacological CBS and 3-MST Inhibition on the Development of ER Stress Response 

**Figure 11:**
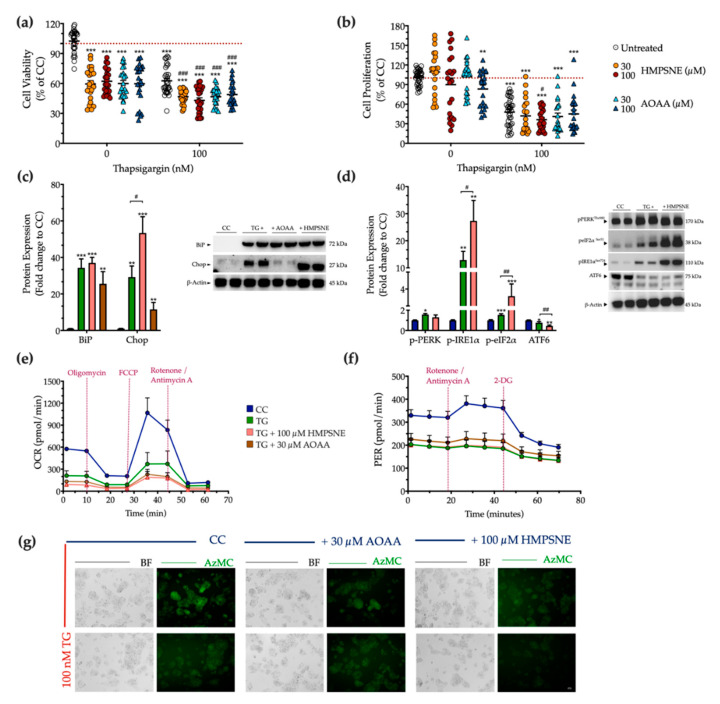
**3-MST pharmacological inhibition strengthens UPR apoptotic signals to exacerbate cell growth arrest under condition of persistent ER stress.** HepG2 cells were serum-starved for 8 h and treated with the vehicle or 100 nM of thapsigargin (TG), in the presence or absence of HMPSNE or AOAA for 16 h. Micro-cultures were assayed for XTT conversion (**a**) and BrdU incorporation (**b**) to assess cell viability and proliferation, respectively. Moreover, cells were harvested and processed for protein expression of the UPR sensors–BiP (**c**), IRE1α (activating phosphorylation at Ser724), PERK (activating phosphorylation at Thr 980), eIF2α (inhibitory phosphorylation at Ser51), and total ATF6 (**d**), along with the UPR apoptotic mediator–Chop (**c**). We also monitored the oxygen consumption rate (OCR) over the sequential injection of oligomycin (1 µM; ATP synthase inhibitor), FCCP (1.5 µM; mitochondrial uncoupler) and of rotenone + antimycin A (0.5 µM; Complex I and III inhibitors) (**e**) and the proton efflux rate (PER) over the sequential injection of rotenone + antimycin A and 2-deoxy-D-glucose (20 mM) (**f**) with the Seahorse XFe24 Extracellular Flux Analyzer for assessing glycolysis and oxidative phosphorylation, respectively. Representative pictures of the AzMC signal were captured with an Olympus CKX53 inverted microscope at 10× magnification; the left sub-panel corresponds to the bright-field channel (BF) for whole-cell imaging, while the right sub-panel to the DAPI fluorescent channel for AzMC imaging (**g**). Each graph line and bar represent mean ± SEM from three independent experiments. Data are expressed as a percentage or fold change of the control (vehicle-treated) conditions (CC). * *p* ≤ 0.05, ** *p* ≤ 0.01 and *** *p* ≤ 0.001 compared to corresponding CC; ^#^ *p* ≤ 0.05, ^##^ *p* ≤ 0.01 and ^###^ *p* ≤ 0.001 compared to the corresponding TG-treated cells.

Next, we examined the physiological effect of the pharmacological inhibition of the 3-MST or CBS/CSE enzymes on cell fate and proteostasis following chronic thapsigargin co-treatment. Pharmacological inhibition of the 3-MST enzyme was achieved by 30 µM and 100 µM HMPSNE (similar to prior studies using these concentrations of the inhibitor, which produce a marked pharmacological inhibitory effect without adversely affecting cell viability [6,17,18,19]). Interestingly, and in contrast to multiple other cell types where these concentrations were not affecting baseline cell function [6,17,18,19]-in HepG2 cells, under normal, non-stressed conditions HMPSNE significantly reduced the XTT conversion, but without affecting BrdU incorporation for cell proliferation, as illustrated in Figure 11a,b. In the presence of chronic thapsigargin exposure, HMPSNE exacerbated the ER-stress-induced suppression of cell viability and proliferation (Figure 11a,b). 

For the combined pharmacological inhibition of CBS and CSE, we used aminooxyacetic acid (AOAA), an agent that is commonly referred to as a “CBS inhibitor”, even though in fact it inhibits both CBS and CSE (as well as a host of additional PLP-dependent enzymes) [20,21]. We have utilized the AOAA concentrations of 30 µM and 100 µM, which have been commonly used in prior studies, and in most cell types do not affect baseline cell viability or cell function (although it can sensitize cells to cytotoxic agents) [22,23,24,25,26,27,28,29,30]. However, in our HepG2 cells, we have noticed that even at the lowest concentration of AOAA, there is a baseline impairment of cell viability and proliferation (Figure 11a,b). AOAA enhanced the ER-stress-associated reduction in the hepatic cell viability, without significantly affecting the suppression of cell proliferation (Figure 11a,b). 

Considering the concentrations in which a significant synergistic effect of thapsigargin and the inhibitor on cell growth arrest, we selected to continue with the concentration of 100 µM of HMPSNE and 30 µM AOAA for our next set of experiments. Both selected concentrations have successfully decreased the intracellular H_2_S levels at basal (control) conditions, as shown in Figure 11g. Neither inhibitors affected the ectopic expression of the UPR sensor-BiP (Figure 11c). Intriguingly, though, HMPSNE doubled the aberrant Chop expression, signifying the exacerbation of UPR apoptotic signals under chronic ER stress (Figure 11c). The latter correlates with a deterioration of the reduced AzMC fluorochrome intensity under conditions of persistent ER stress (Figure 11g). 

With regards to the upstream effectors of the UPR, HMPSNE co-treatment augmented the upregulated activation of IRE1α and the inhibitory phosphorylation of eIF2α, without affecting PERK activity (Figure 11d). Moreover, HMPSNE amplified the decreased expression of the third UPR effector ATF6 (Figure 11d) that altogether may account for the exacerbation of UPR apoptotic outcome. These effects were not accompanied by a deterioration in the mitochondrial respiratory function under conditions of chronic ER stress (Figure 11e,f).

### 3.7. Delayed Supplementation of H_2_S Exerts Partial Effects on the ER Stress Response 

**Figure 12:**
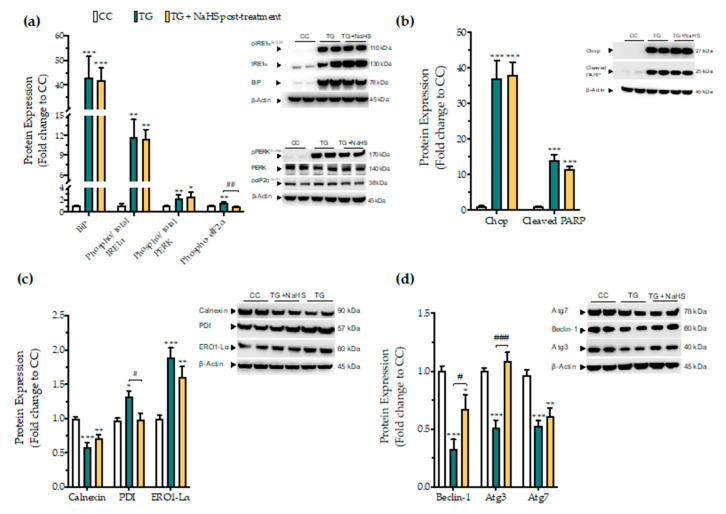
**Delayed exogenous supplementation with H_2_S does not ameliorate UPR activation though it partially rescues autophagy impairments.** HepG2 cells were serum-starved for 8 h and treated with the vehicle or 100 nM thapsigargin (TG) for 16 h. NaHS treatment initiated 4 h post thapsigargin. Next, cells were harvested and the extracted protein was processed for Western blotting analysis of the expression levels of BiP, activating phosphorylation of IRE1α at the serine residue 724 (Ser724), the activating phosphorylation of PERK at the threonine residue 980 (Thr980), the inhibitory phosphorylation of eIF2α at Ser51 (**a**). We additionally quantified the expression levels of the UPR apoptotic mediator Chop and of the cleaved PARP (**b**). Moreover, the expression of the chaperones–calnexin, PDI and ERO1-Lα (**c**), and of the autophagy-related proteins (Atg)-beclin-1, Atg3 and Atg7 (**d**) were assessed. Each bar represents mean ± SEM from three independent experiments. Data are expressed as a fold change of the control (vehicle-treated) conditions (CC). * *p* ≤ 0.05, ** *p* ≤ 0.01; *** *p* ≤ 0.001 compared to CC; ^#^ *p* ≤ 0.05, ^##^ *p* ≤ 0.01, ^###^ *p* ≤ 0.001 compared to TG-treated cells. For the abbreviations used, please refer to the main text.

Next, we tested whether there is a critical time window for the regulatory effects of H_2_S donation on proteostatic processes under conditions of persistent ER stress. NaHS treatment, when delayed to 4 h post thapsigargin did not affect the aberrant expression of the effector proteins of the UPR–BiP, IRE1α, PERK. In addition, it ameliorated the apoptotic signals driven by ectopic Chop expression and PARP cleavage following chronic ER stress (Figure 12a,b). The suppressed expression of the local chaperone calnexin and the up-regulated levels of the oxidoreductase ERO1-Lα remained unaffected (Figure 12c). However, delayed H_2_S administration had an effect on eiF2α activation; it also normalized of the aberrant expression of disulfide-bond formation-related chaperone PDI (Figure 12c). Furthermore, the delayed supplementation of H_2_S partially counteracted the autophagy impairments, evident by the attenuation of the suppressed expression of beclin-1 and Atg3 protein for autophagosome formation; however, it exerted no significant effect on Atg7 (Figure 12d).

### 3.8. Effect of H_2_S on the Tunicamycin-Induced ER Stress Response 

**Figure 13:**
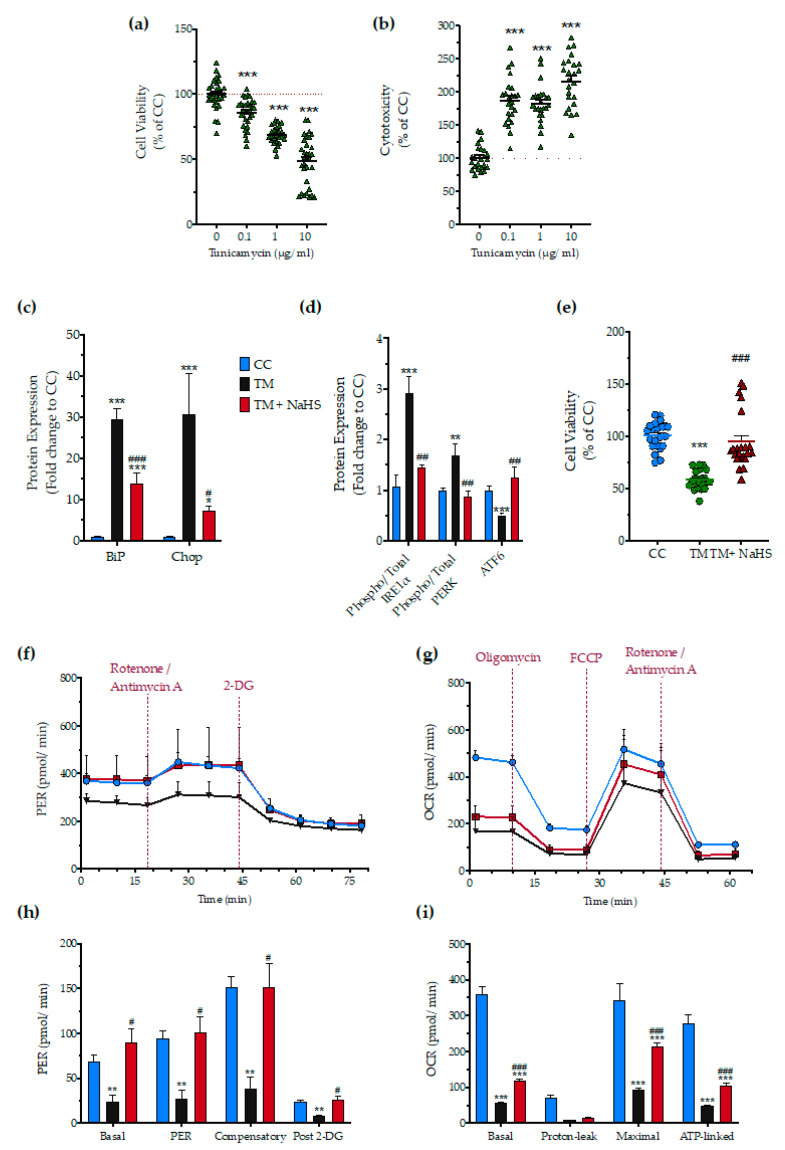
**No-calcium dependency in the hepatoprotective effects of NaHS under conditions of persistent ER stress.** HepG2 cells serum-starved for 8 h and treated with 0, 0.1, 1, and 10 µg/mL of tunicamycin (TM) for 16 h for stressor concentration optimization. Micro-cultures were assayed for the XTT conversion (**a**) to assess cell viability. Cell supernatant was collected and processed for the LDH activity (**b**) to determine cytotoxicity. We selected the concentration of 1 µg/mL of tunicamycin for the experiments. For the subsequent experiments, HepG2 cells were treated with the vehicle or 1 µg/mL of tunicamycin, with/without 100 µM NaHS for 16 h. Following the treatment incubation, whole-cell lysate was collected and processed for protein expression of the UPR sensors-BiP (**d**), IRE1α (activating phosphorylation at Ser724), PERK (activating phosphorylation at Thr 980), and total ATF6 (**c**). We additionally quantified the expression levels of the UPR apoptotic mediator-Chop **(c)**. Micro-cultures were assayed for XTT conversion (**e**) or cellular bioenergetic function using the Seahorse XFe24 Extracellular Flux Analyzer for assessing oxidative phosphorylation (**f**,**h**) and glycolysis (**g**,**i**). Each graph line and bar represent mean ± SEM from three independent experiments. Data are expressed as a percentage or fold change of the control, untreated/unstressed conditions (CC). * *p* ≤ 0.05, ** *p* ≤ 0.01 and *** *p* ≤ 0.001 compared to corresponding CC; ^#^ *p* ≤ 0.05, ^##^ *p* ≤ 0.01 and ^###^ *p* ≤ 0.001 compared to the corresponding TM-treated cells.

Next, we tested whether H_2_S also affects the ER stress response to a different ER stress inducer agent, tunicamycin. After testing the sensitivity of the HepG2 cells to tunicamycin (Figure 13a,b), we selected the 1 µg/mL concentration of the stressor agent for the subsequent studies. NaHS treatment had a significant protective effect against the aberrant expression of the effector proteins of the UPR–BiP, IRE1α, PERK, ATF6, and Chop (Figure 13c,d), and protected against the deterioration of cellular bioenergetics (Figure 13e–i).

## 4. Discussion

Pharmacological disruption of ER-calcium dynamics offers an integrated preclinical in vitro model to investigate the mechanisms and experimental therapy of ER-stress-associated hepatic cell dysfunction. Thapsigargin acts as a specific, non-competitive inhibitor of the Sarco/ER calcium ATPase (SERCA) and precludes the cell from pumping calcium into the sarcoplasmic and endoplasmic reticula that transiently increases the cytosolic calcium levels [31]. The concomitant depletion of ER calcium stores hampers the activity of calcium-dependent local chaperones, like calnexin, and induces an aberrant accumulation of unfolded/misfolded protein within the organelle lumen that defines the ER stress [32]. The cell, in return, engages the UPR network that integrates signals about the chronicity and severity of the insult and coordinates the activation patterns of PERK, ATF6, and IRE1a effector signaling to determine cell fate [14,33]. For instance, prolonged ER stress sustains over time the up-regulated activation of PERK-a Ser/Thr protein kinase of which the catalytic core shares a substantial homology to eIF2A and phosphorylates eiF2α at the serine 51 residue. This phosphorylation impedes the delivery of the initiator methionyl-tRNA to the ribosome and ceases global protein translation. Though an adaptive stress mechanism, at first sight, sustained translational repression downstream of chronic ER stress mediates apoptosis via the persistent ectopic expression of the pro-apoptotic transcription factor Chop (also termed as growth-arrest, DNA-damage 153-GADD153) [34,35].

Chronic ER stress can additionally favor the hyperactivation of IRE1α-a dual function type I transmembrane protein with Ser/Thr protein kinase with endoribonuclease activities. When hyperactivated, IRE1α recruits tumor necrosis factor receptor (TNFR)-associated factor-2) (TRAF2) and apoptosis signaling kinase 1 (ASK1) to activate c-Jun N-terminal kinase (JNK) and nuclear factor κB (NF-κB). It can additionally exacerbate the function of the regulated IRE1-dependent decay (RIDD) pathway to aberrantly chop RNAs for proteins/factors of ER biogenesis and for degradation pathways that altogether increase cell sensitivity to stress and promote apoptosis [35]. Monitoring of multiple ER-stress-related outcome variables, as conducted in the current study, shows that the in vitro model employed in the current experiments has successfully recapitulated the various core aspects of ER stress. Chronic thapsigargin treatment of HepG2 cells, as expected, augmented PERK and IRE1α arm signaling to provoke an ectopic expression of Chop that correlated with a prominent downregulation in calnexin protein levels and an excess in ERO1-Lα and PDI protein expression. The abnormal expression pattern of these chaperones was also associated with suppressed cell bioenergetics and increased mitochondrial oxidative stress. Calnexin silencing has been previously demonstrated to sensitize cardiomyocytes to ER stress and mediates apoptosis through Chop up-regulation and calcium deregulation [36]. Induction of the oxidoreductase ERO1-Lα downstream of Chop perturbs the ER redox state [37], which, in turn, stimulates inositol-1,4,5-trisphosphate receptor (IP3R)-mediated calcium efflux into cytosol [38]. Calcium mishandling could influence multiple pathways upstream of the core apoptosis machinery and, most importantly, mitochondrial membrane polarization and respiration [39]. Overexpression of PDI has triggered the aberrant production of reactive oxygen species and, thus, apoptosis through interacting with and regulating the state of the NAPDH oxidase [40]. Furthermore, the IRE1α/JNK signaling arm activates the ER-resident caspase 12 and imbalances the expression and activity ratio between pro-apoptotic and pro-survival members of the Bcl-2 family, which orchestrates a downstream cascade for PARP degradation and programmed cell-death initiation [35], as reflected in the ‘baseline’ results (i.e., the effect of thapsigargin on the various ER-stress-associated parameters) presented in the current report.

The liver is a fundamental site for endogenous H_2_S biosynthesis and detoxification. Hepatic H_2_S production has been previously implicated in the regulation of hepatic insulin sensitivity, and glucolipid metabolism [41,42]. Aberrant H_2_S production and signaling contribute to a plethora of chronic diseases of this organ, including non-alcoholic steatohepatitis, hepatocellular carcinoma, and liver fibrosis [41,42]. Supplementation of H_2_S (with either NaHS or other classes of H_2_S donors) has been previously shown to suppress liver inflammation, ameliorate intracellular stress responses, and to counteract hepatic injury associated with a variety of insults ranging from ischemia-reperfusion, xenobiotic toxicity, and obesity, diabetes mellitus and metabolic disease (alone or in their various combinations) [41,42,43,44,45,46,47]. 

In the current study, we have tested, in a well-characterized in vitro system, whether exogenous or endogenous H_2_S modulates the ER stress response (as triggered by the pharmacological perturbation of ER calcium homeostasis). The findings clearly demonstrate that supplementation (pharmacological restoration) of H_2_S counteracts the thapsigargin-induced pathophysiological alterations in a comprehensive array of ER-stress-related markers, which, in turn, culminates in an improvement of hepatocyte bioenergetics, viability and proliferation. 

Importantly, H_2_S supplementation was found to ameliorate the abnormal BiP expression along with a partial or complete normalization of PERK, eiF2α, and IRE1α phosphorylation patterns. H_2_S supplementation also mitigated ectopic Chop expression and prevented PARP cleavage, attenuated calnexin suppression, and restored PDI and ERO1-Lα protein levels. These effects were not only associated with an improvement of oxidative phosphorylation and glycolysis, but also with a normalization of mitochondrial superoxide production and a beneficial effect on the ratio of pro-survival to pro-apoptotic Bcl-family members. Collectively, our findings suggest that the H_2_S supplementation can regulate the induction patterns of UPR effectors and pro-apoptotic mediators and promote chaperone homeostasis in the ER lumen. Altogether, these effects improve hepatic cell proteostasis under conditions of persistent ER stress. We hypothesize that the various functional responses (improved bioenergetics and proliferation) are downstream of these responses. The increased mitochondrial superoxide production may either be a cause or a consequence of mitochondrial dysfunction, or these two events may form a positive feedforward pathophysiological cycle under the conditions of ER stress.

Acute SERCA channel inhibition precludes autophagosome formation [48] and fusion with lysosomes [49], resulting in autophagy arrest. Autophagy failure [50] combined with deregulated UPR, may detrimentally affect protein generation and degradation processes that can exhaust the liver regeneration capacity and drive the progression of various liver diseases. Autophagy is a tightly regulated pathway that allows cells to eliminate harmful/damaged components and lipid droplets through catabolism and recycling to maintain nutrient and energy homeostasis. It therefore, constitutes a crucial mechanism for preserving structures and functioning of subcellular organelles, including ER and mitochondria, when operating at basal levels and for cell adaptation and survival upon stress [50]. Several studies have shown that NaHS promotes autophagy and cell survival in the liver, heart, and brain [51]. Accordingly, NaHS has been previously shown to prevent the aberrant inflammation and hepatic injury by halting excessive autophagy in the murine liver [43]. Exacerbated autophagic flux can degrade endogenous inhibitors of apoptosis and Atg components that lead to cell death. That dual role has been further attributed to autophagy under ER stress conditions [52]. Here, we show that apoptotic ER stress correlates with a substantial decrease in the protein levels of beclin-1, Atg3, and Atg7 that corresponds to a suppressed autophagosome formation intracellularly. This deficiency in autophagy-related components may stem from the ectopic Chop expression that can limit the transcriptional control of a dozen of Atg genes involved in phagophore elongation and maturation into the autophagosome as part of its apoptotic mechanism of action upon persistent ER stress [53,54]. Strikingly, NaHS co-treatment ameliorates Atg3, Atg7, beclin-1, and reinstates hepatic autophagy that signals for cell adaptation and survival upon unmitigated ER stress.

The effect of H_2_S donation is clearly time-dependent. Delaying the administration of the H_2_S donor to 4 hours after the start of thapsigargin exposure of the HepG2 cells resulted in a loss of most of the protective effects. These findings point towards the hypothesis that the site for the beneficial action of H_2_S lies in early events of the intracellular stress cascade. Perhaps, it is at the level of the SERCA channel inhibition (the activity of SERCA was not directly measured in the current study), or at the level of the consequent calcium mobilization. However, the direct modulation of the SERCA channel or ER calcium levels by H_2_S seem less likely. Co-treatment with NaHS also exerted beneficial effects against the tunicamycin-induced ER stress. Tunicamycin, another well-documented ER-stress inducer, inhibits protein N-glycosylation in the ER and thus increases the unfolded protein load within the organelle lumen. It potentiates a hepatic ER stress response that is phenotypically similar to the one triggered by thapsigargin, though considered a “calcium-independent” one [55,56]. Considering these, possible candidates for the mechanism of action of H_2_S may be those (relatively early) cellular effectors or processes that regulate the activation of the three main proximal effectors of UPR (PERK, IRE1α, and ATF6) that are common to both calcium-dependent and -independent triggers of ER stress. Multiple prior reports in the literature have indicated that H_2_S donation can ameliorate various ER-stress-related downstream parameters (in signaling and/or in cellular bioenergetics or viability) [57,58,59,60,61,62,63,64,65]. However, the exact molecular site(s) of the beneficial action of H_2_S on the initiation and progression of ER stress and ER-stress-associated cellular responses remain to be further refined. In this respect, the partial efficacy of delayed H_2_S administration on some (but not all) ER-stress-related parameters may be helpful for us to re-focus on some potentially relevant pathways with prolonged activation during ER stress. Although the UPR effector kinases, PERK and IRE1α remained unaffected by delayed H_2_S administration, a major component of the integrated stress response, the phosphorylation of eIF2α, was, nevertheless, still inhibited. H_2_S-induced regulation of eIF2α phosphorylation patterns may be potentially linked to the regulation of GCN2 kinase [66,67] and to the persulfidation status of the catalytic subunit of protein phosphatase (PP1c) that performs the dephosphorylation of eIF2α [68].

In contrast to the clear ameliorative effect of exogenous H_2_S on a host of ER-stress-related parameters, pharmacological inhibition of CBS/CSE and/or pharmacological inhibition or partial silencing of 3-MST (to suppress the endogenous production of H_2_S) exerted a less complete effect on the ER-stress responses. As expected, on some (but not all) parameters, an exacerbation of the ER-stress-responses was noted, which is consistent with some of the published literature [69,70]. These findings are also consistent with the hypothesis that endogenously produced H_2_S may, indeed, exert some protective effect against the pathophysiological events associated with ER stress response. We hypothesize that a possible reason for the partial/incomplete effects seen after silencing/inhibition of individual H_2_S producing enzymes is that endogenous H_2_S production is the result of multiple enzymatic reactions (as well as various non-enzymatic processes); thus, inhibition of any individual enzymatic source of H_2_S (e.g., inhibition of 3-MST or partial genetic knockdown of 3-MST) will reduce (but will not completely eliminate) endogenous H_2_S production. 

Interestingly, HMPSNE does not affect BiP, of which the induction not only serves as a signal for ER stress but also represents the “adaptive” pro-survival component of the UPR to cope with the unfolded protein load with the ER lumen. HMPSNE does exacerbate the ectopic Chop expression that correlates with an amplification of cell growth arrest under conditions of chronic ER stress. This finding signifies that the exogenous inhibition of the 3-MST/H_2_S system activity selectively amplifies the apoptotic signals of the UPR to create “a-point-of-no-return” for cell fate. Furthermore, our data show that HMPSNE amplifies Chop by boosting eIF2α/ATF4 arm without altering PERK activity. As discussed earlier, the GCN2 kinase for eIF2α phosphorylation at serine 51 has been implicated in the signaling repertoire modulated by H_2_S [66] that may explain our biochemical phenotype following HMPSNE treatment. In addition to the eIF2α phosphorylation, HMPSNE strengthens the IRE1α hyperactivation and exacerbates the suppressed expression of ATF6. In vivo, this combinatorial deregulation of these two UPR arms seems to drive the aberrant lipid metabolism and the progression of liver steatosis [71,72,73]. 

## 5. Conclusions

**Figure 14:**
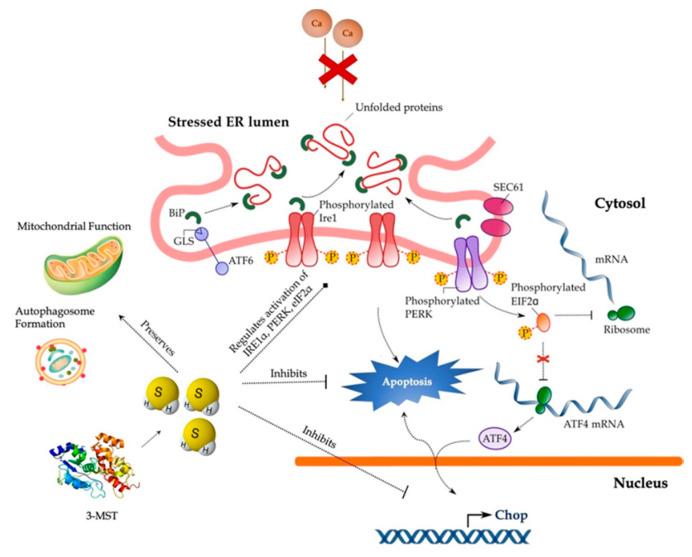
**Cytoprotective effects of H_2_S during ER stress in hepatocytes.** Persistent SERCA channel inhibition by thapsigargin provokes aberrant activation of the UPR effectors–PERK, ATF6, and IRE1α–that results in the ectopic expression of the pro-apoptotic Chop, autophagic arrest, bioenergetic crisis, and ultimately, cell death. Restoration of H_2_S levels resolves the “terminal UPR” and preserves mitochondrial function and autophagosome formation to swift hepatic cell fate towards survival. For further mechanistic details, please see the main text.

In conclusion, our findings implicate the 3-MST/H_2_S system in the intracellular network that governs proteostasis and hepatic ER stress adaptability (Figure 14) and thus reinforce the therapeutic potential of pharmacological H_2_S supplementation. Despite this exciting pharmacological direction raised by the present study, the immortalizing nature and the limited drug-metabolism capabilities of HepG2 cells pose a considerable burden for translating these data into the human liver disease. Consequent proof-of-concept studies in primary hepatocytes will enlighten us on the specificity of the 3-MST/H_2_S system for determining UPR outcome for hepatic cell fate. 

## Figures and Tables

**Table 1:** List of primary antibodies used in Western blotting. (SM = skimmed milk; BSA: bovine serum albumin).

Target	Blocking Buffer	Dilution	Isotype	Manufacturer	Catalogue Number
*β*-Actin	5% *w*/*v* SM	1:2000	Mouse IgG	Cell Signaling	3700
Beclin-1	5% *w*/*v* SM	1:1000	Rabbit IgG	Cell Signaling	3495
Atg3	5% *w*/*v* SM	1:1000	Rabbit IgG	Cell Signaling	3415
Atg7	5% *w*/*v* SM	1:1000	Rabbit IgG	Cell Signaling	8558
ATF6	5% *w*/*v* SM	1:1000	Mouse IgG	Abcam	ab122897
BiP	5% *w*/*v* SM	1:1000	Rabbit IgG	Cell Signaling	9956
Calnexin	5% *w*/*v* SM	1:1000	Rabbit IgG	Cell Signaling	9956
PDI	5% *w*/*v* SM	1:1000	Rabbit IgG	Cell Signaling	9956
Ero1-L*α*	5% *w*/*v* SM	1:1000	Rabbit IgG	Cell Signaling	9956
Chop	5% *w*/*v* SM	1:1000	Mouse IgG	Cell Signaling	9956
IRE1*α*	5% *w*/*v* SM	1:1000	Rabbit IgG	Cell Signaling	9956
Phospho-IRE1 (Ser724)	5% *w*/*v* SM	1:1000	Rabbit IgG	Abcam	ab48187
PERK	5% *w*/*v* SM	1:1000	Rabbit IgG	Cell Signaling	3192
Phospho-PERK (Thr980)	5% *w*/*v* BSA	1:1000	Rabbit IgG	Thermo Fisher	15033
Phospho-eIF2α (Ser51)	5% *w*/*v* BSA	1:1000	Rabbit IgG	Cell Signaling	9721
Bak	5% *w*/*v* SM	1:1000	Rabbit IgG	Cell Signaling	12105
Bcl-xL	5% *w*/*v* SM	1:1000	Rabbit IgG	Cell Signaling	2764
Cleaved PARP	5% *w*/*v* SM	1:500	Rabbit IgG	Abcam	ab32064
CBS	5% *w*/*v* SM	1:1000	Rabbit IgG	Cell Signaling	14782
3-MST	5% *w*/*v* SM	1:1000	Rabbit IgG	Abcam	ab154514
CSE	5% *w*/*v* SM	1:1000	Rabbit IgG	Abcam	ab151769
TST	5% *w*/*v* SM	1:1000	Rabbit IgG	Abcam	ab166625
ETHE1	5% *w*/*v* SM	1:1000	Rabbit IgG	Abcam	ab174302
